# Clinical reporting for personalized cancer genomics requires extensive access to subscription-only literature

**DOI:** 10.5195/jmla.2023.1572

**Published:** 2023-04-21

**Authors:** Schnell D'Souza, Gregory Downs, Shawn Hendrikx, Rouhi Fazelzad, Gabriel Boldt, Karen Burns, Darlene Chapman, Declan Dawes, Antonia Giannarakos, Lori Anne Oja, Risa Schorr, Maureen Babb, Amanda Hodgson, Jessica McEwan, Pamela Jacobs, Tracy Stockley, Tim Tripp, Ian King

**Affiliations:** 1 dsouzas@uoguelph.ca, University of Guelph, Guelph, ON, Canada.; 2 Gregory.downs@uhnresearch.ca, University Health Network, Toronto, ON, Canada.; 3 shendri4@uwo.ca, Collections and Content Strategies Librarian, Western University, London, ON, Canada.; 4 rouhi.fazelzad@uhn.ca, Information Specialist, Library and Information Services, University Health Network, Toronto, ON, Canada.; 5 gabriel.boldt@lhsc.on.ca, Clinical Librarian, London Health Sciences Centre, London Regional Cancer Program, London, ON, Canada.; 6 karen.burns@uhn.ca, University Health Network, Toronto, ON, Canada.; 7 darlene.chapman@iwk.nshealth.ca, Operations Manager, Library Services, IWK Health, Halifax, NS, Canada.; 8 declan.dawes@mail.utoronto.ca, University of Toronto, Toronto, ON, Canada.; 9 antonia.giannarakos@thp.ca, Senior Librarian, Library & Knowledge Services Canada, Trillium Health Partners, Mississauga, ON, Canada.; 10 lori.oja@utoronto.ca, Executive Director HSICT, Health Sciences Information Consortium of Toronto, Toronto, ON, Canada.; 11 rshorr@toh.ca, The Ottawa Hospital, Ottawa, ON, Canada.; 12 maureen.babb@umanitoba.ca, Science Liaison Librarian, University of Manitoba, Winnipeg, MB, Canada.; 13 amandah@cadth.ca, University of Ottawa, Ottawa, ON, Canada.; 14 jessica.mcewan@uottawa.ca, User Experience Librarian, University of Ottawa, Ottawa, ON, Canada.; 15 pjacobs@uoguelph.ca, University of Guelph, Guelph, ON, Canada.; 16 Tracy.Stockley@uhn.ca, University Health Network, Toronto, ON, Canada.; 17 tim.tripp@uhn.ca, Director, Library & Information Services, University Health Network, Toronto, ON, Canada.; 18 ian.king@uhn.ca, University Health Network, Toronto, ON, Canada.

**Keywords:** Cancer, genetics, open access, university collections, health sciences library collections, subscription access, precision medicine, pathology, oncology

## Abstract

**Objective:**

Medical care for cancer is increasingly directed by genomic laboratory testing for alterations in the tumor genome that are significant for diagnosis, prognosis and therapy. Uniquely in medicine, providers must search the biomedical literature for each patient to determine the clinical significance of these alterations. Access to published scientific literature is frequently subject to high fees, with access limited to institutional subscriptions. We sought to investigate the degree to which the scientific literature is accessible to clinical cancer genomics providers, and the potential role of university and hospital system libraries in information access for cancer care.

**Methods:**

We identified 265 journals that were accessed during the interpretation and reporting of clinical test results from 1,842 cancer patients at the University Health Network (Toronto, Canada). We determined the degree of open access for this set of clinically important literature, and for any journals not available through open access we surveyed subscription access at seven academic hospital systems and at their affiliated universities.

**Results:**

This study found that nearly half (116/265) of journals have open access mandates that make articles freely available within one year of release. For the remaining subscription access journals, universities provided a uniformly high level of access, but access available through hospital system collections varied widely.

**Conclusion:**

This study highlights the importance of different modes of access to the use of the scientific literature in clinical practice and points to challenges that must be overcome as genomic medicine grows in scale and complexity.

## INTRODUCTION

Routine care for many cancers now includes testing to identify alterations in the tumor genome that drive the patient's disease [1]. In this paradigm of “personalized” or “precision” medicine, physicians use genomic testing results to diagnose disease type, determine the patient's prognosis, and to select efficacious therapy, often therapy targeted to a particular molecular alteration present in the tumor [2,][3]. Because cancer is a heterogeneous disease, each patient's tumor will have a particular and often unique combination of genomic alterations, and patients with the same cancer type may receive very different treatments based on their tumor's genomic profile. As more knowledge has been gained about the genome, and more specifically targeted drugs have been developed, genomic biomarkers testing has increasingly become the standard of care, and the number of patients receiving genomic tumor sequencing and the number of genes that are routinely sequenced has increased rapidly [1,4–6].

Genomic tumor sequencing results are conveyed to physicians and patients mainly through written reports. Certified clinical laboratories performing genomic testing issue reports identify the genomic alterations present in a tumor sample, and also provide an interpretation that comments not only on the technical parameters of the testing but also on the clinical implications of the results. Because of the complexity of the genome, reporting standards have evolved to require that laboratories give an interpretation of the significance of each variant reported [7] (See Appendix A). Clinical reports must distinguish between alterations that have a known functional or clinical significance and those that are known to be benign, and importantly, must also identify alterations whose significance is unknown due to lack of evidence [7].

As a result, clinical genomics reports often contain, by necessity, personally focused, patient-specific reviews of the scientific literature. Each alteration is researched by the reporting laboratory, and an interpretation is issued based on information found in the scientific and medical literature. Literature searches are accomplished using a combination of manual search using conventional literature search tools (e.g., Google, Google Scholar, PubMed/MEDLINE), and by using proprietary and publicly available cancer knowledgebases, databases that contain information on the impacts of particular genomic alterations on protein function and therapy which can be searched either manually or automatically via an application-programming interface [9–13]. Because many of the genomic alterations identified in genomic testing are individually quite rare, information that is clinically significant for a patient is frequently found in small cohort studies or individual case reports, often published in highly specialized journals.

Genomic interpretation is generated and consumed by medical personnel of varying levels of training. Once issued, clinical reports are read and used by many care providers, including specialist physicians, generalist physicians, nurses, and genetic counselors, and ultimately are available to patients, who are increasingly willing and able to research their genomic test results [14,15].

As personalized medicine has developed and expanded, the need for access to scientific literature has increasingly become an important issue. Genomic testing has, until recently, been carried out largely as part of research studies at medical centers affiliated with universities, a setting where those generating and consuming genomic interpretations have enjoyed extensive access to the scientific literature through university library subscriptions. However, as genomic testing has moved increasingly into standard care, it has involved more and more providers in non-academic roles that do not have extensive access to the scientific literature needed for genomic interpretation. Within some academic centers, personnel who do not have university affiliations rely heavily on library collections kept by hospitals for literature access. At the same time that cancer genomic testing has expanded, obtaining access to the scientific literature required for interpretation of these results has become more complex and expensive for public institutions [16–18].

To assess how well current systems meet the needs of precision medicine, we investigated the accessibility of literature used by a clinical laboratory in the interpretation of genomics results. We compiled the journal article references accessed during the interpretation of genomic testing results for nearly 1000 lung cancer patients and 1000 patients with hematopoietic malignancies, incorporating results generated using both manual search and by IBM Watson for Genomics, an automated system. Further, we surveyed medical centers and university libraries in Canada to assess how well the subscription-only sources were covered by these institutional collections.

## METHODS

### Institutional Review

The research in this study was approved by the Research Ethics Board of the University Health Network (Study ID 18-5808).

### Dataset of Clinically Accessed PubMed Journal Articles

The test dataset consisted of PubMed IDs (PMIDs) of journal articles that were accessed and recorded for clinical interpretation of sequence variants in two settings: first, in routine biomarker testing for a series of non-small cell lung cancer cases tested at the UHN Genome Diagnostics Lab (lung cancer series), second, a series of cases tested as part of the AGILE study of genomic profiling of hematological malignancies (leukemia series). For the lung cancer series, testing was performed between 2015 and 2019, and the leukemia case series testing was performed between 2014 and 2017. Lung cancer testing was performed on 15 genes that are clinically important for lung cancer. Leukemia testing analyzed 54 genes used for diagnosis, risk evaluation, and therapy selection in hematological disorders. For both patient series, PMIDs were extracted from curation notes recorded in the Alissa genomics analysis platform (Agilent) using custom Python scripts. In parallel, cases from both series were analyzed using the Watson for Genomics platform (IBM). Per system requirements, sequencing results in the form of a Variant Call Format file (.vcf) were input, along with an appropriate disease code from the NCI Thesaurus. Interpretation text was outputted in JSON files, which were parsed for PMIDs cited in the interpretive text using custom Python scripts (Appendix 2).

PMIDs were matched to journals using a custom Python script to query the PubMed Application Programming Interface. The National Library of Medicine (NLM) Catalog was used to assign journals to publishers and to link print and electronic International Standard Serial Numbers (ISSNs) for each journal. Clarivate Analytics' Web of Science Core Collection was used to assign one or more subject categories for each journal. Clarivate Analytics' 2019 Journal Citation Reports was used to find and assign the 2019 Impact Factor score for each journal.

### Survey of Open Access Journals

Journals' open access policies were determined using information from the Directory of Open Access Journals, PubMed Central, and from the publisher's website for individual journals. Journals were categorized as Gold Open Access (GOA), Delayed Open Access (DOA), Hybrid Open Access (HOA) or Subscription Only (SO) where information was available. GOA journals have all the articles published in their final peer-reviewed form freely available through the journal or publisher's website on the day of release. Journals were only classified as GOA if it was specifically stated in one of the sources listed above. To maintain congruency with the Public Access Policy of the National Institutes of Health [19], it was decided that any journals that made their articles accessible without restriction any time between 1 day and 12 months after release were classified as DOA. DOA status was confirmed empirically by attempting to access at least two articles published before May 2019 (one year prior to when the study was conducted) for each DOA title. SO refers to journals that do not have a default GOA or DOA mandate (within one year). As a result, a subscription or individual article purchase is required to access necessary information. These journals may also have an HOA policy, an increasingly popular option where authors can elect to publish their articles as open access for a fee [20]. It is not possible to measure the true impact of HOA at the journal level as HOA can vary from article to article.

### Survey of Journal Access at Hospital and University Libraries

The survey of literature access targeted libraries of hospitals, health authorities, and universities that conducts research in medicine. Between May and August 2020, 21 hospital system libraries, 12 university libraries, 10 regional and provincial health authorities, and 4 health library associations were contacted to gauge interest in participation. Seven hospitals and their associated universities agreed to collaborate in this study. The academic hospital systems that collaborated in the study were: University Health Network (UHN), Toronto, ON; Trillium Health Partners (THP), Mississauga, ON; McGill University Health Centre (MUHC), Montreal, QC; The Ottawa Hospital (TOH), Ottawa, ON; IWK Health Centre (IWK), Halifax, NS; London Health Science Centre including The London Regional Cancer Program (LHSC), London, ON, and the Winnipeg Regional Health Authority (WRHA), Winnipeg, MB. For comparison, the collections of the universities with which these hospitals are associated were also analyzed: The University of Toronto (U of Toronto), Toronto, ON - associated with UHN and THP; McGill University, Montreal, QC - associated with MUHC; The University of Ottawa (U of Ottawa), Ottawa, ON – associated with TOH; The University of Manitoba (U of Manitoba), Winnipeg, MB - associated with WRHA; Dalhousie University (Dalhousie), Halifax, NS - associated with IWK Health Centre; Western University (Western U), London, ON - associated with LHSC.

Because information on institutional holdings is complex, methods for collecting holdings lists were unique to each institution. No single survey method was used for all institutions – holdings data were gathered from internal records which were not available publicly. For health science libraries without web-based catalogs of journal holdings (UHN, THP, MUHC, TOH, IWK, LHSC), journal lists from subscription packages relevant to life sciences and medicine were compiled and duplicates merged based on ISSN number. For larger institutions (WRHA, U of T, McGill U., U. of Ottawa, U. of Manitoba, Western U., Dalhousie), holdings were available via public web-accessible catalogs. For these institutions the list of subscription-only journals used in clinical reporting and analysis was searched directly against the public holdings record. Searches were made by ISSN number. All journal holdings lists for libraries were received or created between May 2020 and August 2020.

Statistics on Universities were taken from public informational websites, accessed October 29, 2021 (U of Toronto [21]; Western U [22]; McGill University [23]; U of Manitoba [24]; U of Ottawa [25]; Dalhousie [26]). Library budget figures for universities were determined using Canadian Association of Research Libraries 2018-2019 statistics on ‘Total Library Materials', which accounts for any ‘one-off' purchases as well as ongoing purchases (published in 2022) [27]. Details of embargoes and time gaps in subscriptions could not be obtained from some institutions. Therefore, unless it was specifically determined otherwise, it is assumed that subscriptions include access to the entire back catalog of the journal. When print journal information was provided, it was also assumed that it included the entire back catalog of the journal. Because this assumption is made, the access results obtained represent an upper bound, and may overestimate the extent to which journal articles are accessible.

## RESULTS

### Literature Used in Interpretation of Genomics Test Results

As part of a study into the use of automated systems to provide interpretation for clinical cancer genomics testing, we compiled a dataset of interpretive information generated for a series of 843 lung cancer cases and 999 leukemia cases from the clinical genomics labs at the University Health Network in Toronto, Canada, the largest cancer genetics labs in Canada. For lung cancer patients, sequence variants in 15 genes were analyzed and reported as part of routine clinical biomarker testing over 5 years. For leukemia patients, clinical reports were issued for a panel of 54 genes with value for risk determination and therapy selection in hematological cancers as part of a clinical research study. Genomic variants identified in testing were researched by an analyst group of doctoral-level geneticists and clinical reports issued into patients' medical records. The PubMed ID numbers (PMIDs) of journal articles used in the interpretation of the genomics results were recorded as part of the routine clinical analysis work, and these PMIDs were compiled to create a dataset of references used in this standard manual curation process. Subsequent to this manual curation, genomics test results were analyzed using IBM Watson for Genomics (Watson), an automated system for generating interpretation of the biological and clinical significance of genomics results [28–30]. The Watson system uses natural language processing to query the scientific literature for information about specific genomic alterations and outputs interpretations that contain reference PMIDs. We used these automated interpretations as a second complementary dataset of references.

In total, the clinical interpretation of the results from these case series yielded 9565 article uses, referencing 2264 separate PMIDs (Table 1). For both lung cancer and leukemia results, manual curation yielded a larger number of overall PMID citations than automated curation (see “Total PMID Citations”). However, the automated Watson system produced many more unique citations in its interpretations and drew on more journals (see “Unique PMID Citations”), which may reflect both greater efficiency of automated curation and a broader scope of interpretation designed for general use in multiple settings. Because the leukemia test covered more genes and produced more genomic alterations for analysis, more references were generated by both manual and automated curation for the leukemia dataset.

**Table 1:** Summary of Literature Used in Clinical Interpretation for Lung Cancer and Leukemia

	Leukemia		Lung		Combined Total
	Watson	Manual	Watson	Manual	
Journals	245	55	192	51	265
Publishers	59	22	49	21	65
Total PMID Citations	1735	2851	1491	3488	9565
Unique PMID Citations	1153	243	747	121	

**Figure 1:**
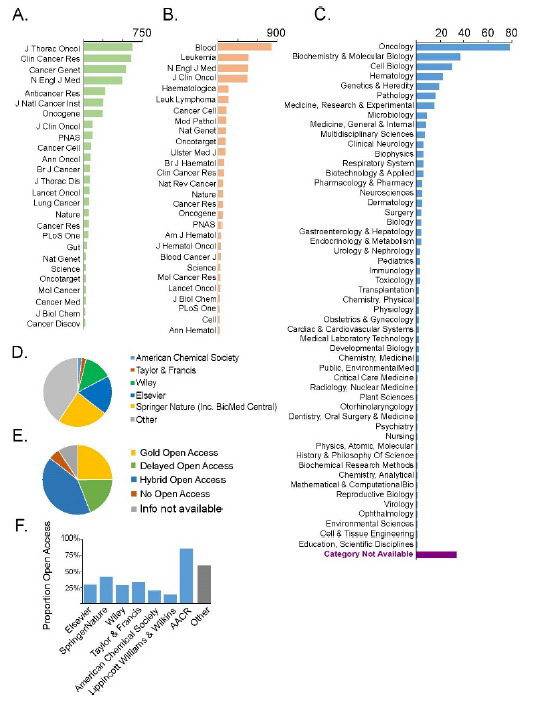
Journals required for interpretation of clinical cancer genomic testing results for a large patient series.

The references in the dataset were derived from 265 different journals, published by 65 different publishers (Table 1, Supplementary Tables 1,2). The journals referenced in clinical interpretation for both lung cancer and leukemia ranged considerably in subject and scope. In general, the most frequently used journals did not have a disease-specific focus, though the most cited journal in the lung cancer dataset (*Journal of Thoracic Oncology*) and the two most cited in the leukemia dataset (*Blood, Leukemia*) are prominent publications focused on those diseases (Fig. 1a, b, Supplementary Tables 1,2). While general interest journals were cited often (e.g., *New England Journal of Medicine, Nature, Science*), publications that have a focus more specifically on cancer genetics were also frequently cited (e.g., *Oncotarget, Oncogene*) (Fig. 1a, b, Supplemental Tables 1,2). Notably, journals were frequently used to interpret a single or small number of cases. Roughly half of the journals in the datasets were cited only twice or once (99/200 lung, 153/254 leukemia) (Supplementary Tables 1,2). Using Clarivate Web of Science (WOS) subject categories, we examined the journal subject areas most often used in clinical interpretation. While Oncology journals were the most commonly used category, many basic science and medical specialty categories were represented (Fig. 1c). Journals that did not have a WOS classification also accounted for a substantial portion of the publications used (34/265 journals).

Journals that were most commonly identified as sources for interpretation and reporting of clinical results for (A) lung cancer or (B) hematological malignancies. Journals cited 25 or more times are shown. For a complete list of journals, see Supplementary Information). (C) Web of Science subject categories for journals in the combined clinical interpretation dataset. (D) Proportion of journals used for clinical interpretation published by large publishers. (E) Proportion of journals used for clinical interpretation by open access policy. (F) Proportion of Gold or Delayed open access journals for publishers well represented in the clinical interpretation dataset. Proportion is given for publishers with 5 or more journals identified as interpretation sources. Other gives the proportion of open access journals in all others not published by the named publishers.

We found that more than half of the journals used in clinical interpretation were published by large publishers – specifically, the five publishers that control the largest share of the Natural and Medical Sciences publishing structure space [25]. The publishers: Springer Nature, Elsevier, Wiley, Taylor & Francis, and the American Chemical Society – accounted for 59% of the journals in our clinical interpretation dataset (Fig. 1d). This was similar to the proportion of journals that these five publishers account for in the NMS database as a whole (53%) in Lariviere et al.'s 2015 paper [31], suggesting that the publications used for clinical interpretation are likely not disproportionally from large or small publishers.

### Impact of Open Access Policies

Because open access policies are becoming increasingly necessary to access the literature for all scientific fields, we surveyed the extent to which journals needed for clinical genomics interpretation were available through open access policies. We researched the open access policies of the journals in the clinical interpretation dataset and categorized them as GOA, DOA, HOA, or SO. We found that fully accessible journals were the minority in the clinical dataset. 50 out of 265 journals (19%) were classified as Gold Open Access and 25% (66/265) had Delayed Open Access policies (Fig. 1e). A further 42% (111/265) had a Hybrid Open Access option in some form, meaning that open access was possible for at least 44% and potentially as much as 85% of articles needed for clinical interpretation. Only 14 journals (5%) had no open access option at all, while for 24 journals (9%) open access policies could not be determined, and both sets were treated as subscription-only.

Journals restricted to subscription-only access more commonly belonged to large publishers. Among publishers with more than 5 journals in the dataset, we found that the proportion of Gold or Delayed Open Access journals was markedly lower for large for-profit publishers than for the remainder of the dataset as a whole (Fig. 1f). Two not-for-profit society publishers were also among these well-represented publishers, and these publishers had different open access rates: journals published by the American Chemical Society (ACS) had a low rate of open access, while the American Association for Cancer Research (AACR) had a high rate of open access. In general, however, large publishers appear to lag in open access rates.

### Journal Subscription Access Survey

For the sizeable number of articles that are not available via open access policies, providers must rely on subscription access. To fully understand the level of access that healthcare providers have to the literature needed to interpret clinical genomics results, we conducted a survey of subscription access at 7 major hospital systems in Canada and 6 of their affiliated universities. All of these hospital systems are engaged in both treatment of patients using genomic biomarker testing and in laboratory testing reporting interpretation of genomics results. As in the United States, physicians and many non-physician staff at these academic hospitals have faculty appointments or other affiliations with universities and are able to access university library collections in their day-to-day work.

All of the six universities surveyed have medical schools. Robust collections in the health sciences are tied to a university needing to support a medical school and the affiliated status of the hospitals around that school are related to the learning requirements of those medical students. These universities predominantly ranged in size between 20,000 and 40,000 students (Table 2), comparable to large public universities in the US. The University of Toronto, which operates three campuses as well as campuses abroad, is a larger system with more than 95,000 students. Library budgets for these universities were comparable across institutions, with similar ratios of budget to student body size (Table 2). In all cases for which estimates are available, university library budgets were considerably larger than those for their affiliated hospitals (Table 2, Table 3). Because these university library systems are large and complex, and because holdings are constantly changing, we were not able to make an estimate of the collection sizes of these institutions' libraries.

**Table 2:** Sizes and library resources of surveyed universities

University	Library Budget (CAD)* (Total Library Materials)	Students	Faculty
University of Toronto	$41,076,582	95,055	15,029
Western University	$15,235,735	31,171	1,325
McGill University	$22,267,442	39,736	1,730
University of Manitoba	$14,131,670	31,020	5,730
University of Ottawa	$15,559,451	44,729	1,223
Dalhousie University	$9,453,291	20,000	999

* Based on Canadian Association of Research Libraries (CARL Statistics), 2018/19 [27]

**Table 3:** Sizes and library resources of surveyed health centers

Hospital/Hospital System	Number of Employees	Holdings (journals)	Budget (CAD)**
University Health Network	16,000	4,713	N/A
London Health Sciences Centre*	15,000	55	N/A
Winnipeg Regional Health Authority	14,000	N/A	$2,000,000 - overall budget $1,000,000 - collections
McGill University Health Centre	12,000	4,682	N/A
The Ottawa Hospital	10,000	8,057	$231,000
Trillium Health Partners	10,800	6,692	$130,000 - journal subscriptions
IWK	4,000	3700	$105,000 – journal subscriptions

* Including London Regional Cancer Program

** Based on budgets directly provided by librarians

N/A not available

All of the hospital systems we surveyed have institutional health sciences library collections which are accessible to all staff involved in clinical genomics. The hospital systems surveyed all employ between 10,000 and 16,000 staff (Table 3). These institutions are also of comparable size to counterpart academic hospitals in the US [32]. We observed that hospital system collections varied greatly in scope (Table 3) and that the size of institutional collections varied widely between hospital systems. Many of the institutions surveyed maintain large collections of titles, numbering in the thousands, but institutions also may maintain only a small collection – opting to instead purchase individual articles when required.

**Figure 2:**
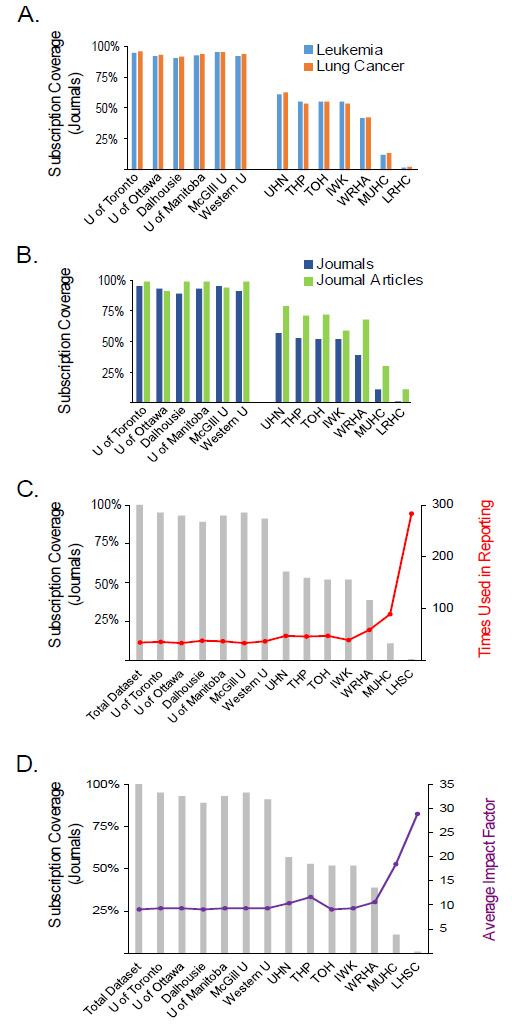
Survey of university and hospital system subscription coverage for literature used in clinical cancer genomics. (A) Proportion of subscription-access journals in the leukemia and lung cancer clinical interpretation datasets that were covered by the holdings of university and health science center libraries surveyed. (B) Proportion of journals and journal articles from subscription-access journals in the combined clinical interpretation dataset covered by these collections. (C) Ratio of the number of instances in the combined clinical interpretation dataset to the number of journals covered by each institutional collection (red line). (D) The average impact factor of journals in each institutional collection (purple line). Proportion of the total number of journals covered by subscription access is shown for comparison (gray bars).

We investigated whether these university collections and hospital system collections were sufficient to provide access to the full set of references used in our clinical interpretation dataset by checking subscription access for all journals which did not have complete public open access (Gold or Delayed Open Access). The collections of all six universities provided almost complete access, with all collections providing access to near or greater than 90% of journals in the dataset (Fig 2a, b). Coverage rates were high both for the journals used in lung cancer cases and the journals used in leukemia cases (Fig. 2a). Coverage rate was also similarly high when subscription coverage was measured in terms of number of uses from the subscribed journals rather than number of journals used, indicating that university collections did not have coverage gaps in journals that are of high value for genomics (Fig. 2b).

In keeping with their smaller collection sizes, we found that coverage rates were significantly less for health science center library collections. The larger collections kept by UHN, THP, TOH and IWK achieved 40-50% coverage (Table 3, Fig. 2a). However smaller collections provided only minimal coverage for clinical genomics (Table 3, Fig. 2a). As with university collections, no difference was seen in the coverage of the journals needed for lung cancer and leukemia interpretation (Fig. 2a). We observed that coverage was somewhat higher in hospital system collections when measured by articles used from the subscribed journals than when measured by number of journals (Fig. 2b). This likely reflects hospital system collections focusing on more general interest publications, or priorities that do not overlap with cancer genomics. We found that the ratio of articles covered to journals covered was much higher for institutions with smaller subscription holdings (Fig. 2c), indicating that their collections are focused on publications with more general use. Similarly, the average impact factor of journals in smaller holdings was also much higher, indicating a focus on general interest journals (Fig. 2d).

**Figure 3:**
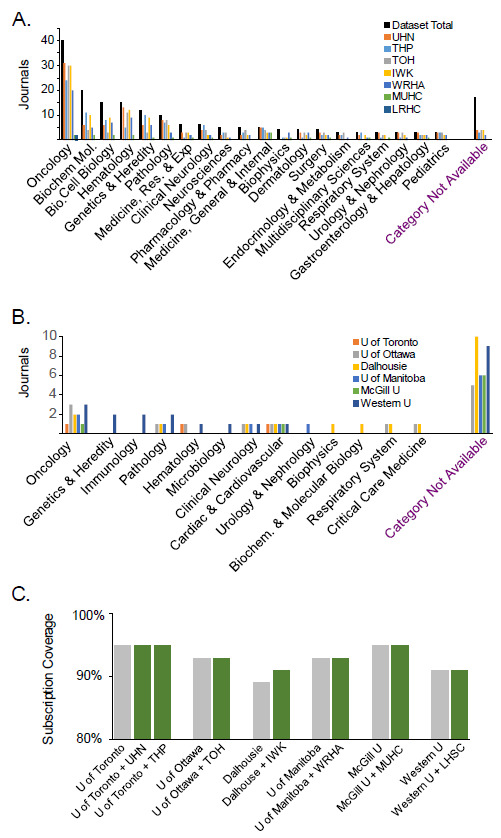
Overlap between university and hospital system library collections. (A) Holdings of surveyed hospital system libraries, grouped by Web of Science category. (B) Web of Science categories for subscription-access journals that were not covered by university holdings, by institution. (C) Additive coverage for university collections, combined with affiliated hospital system collections. Percent coverage is shown for the university system alone (gray), and for the combined university and hospital collections (green).

Because precision medicine often involves interpretation of genomic alterations that are rare or unique, it relies heavily on more focused case reports and small studies likely to be found in very specialized publications. Because hospital library collections may potentially supplement areas of coverage where university collections are weaker, we asked whether these collections were complementary to university collections for clinical genomics or overlapping. To assess the gaps in university holdings, we analyzed WOS categories for the journals in the clinical interpretation dataset that were missing from university collections (Fig. 3a). We found that a high proportion of the gap was in journals that do not have a WOS category, journals that are either highly specialized or were non-English language publications. When we examined the distribution of WOS categories in hospital collections we found that the distribution of categories roughly reflected the dataset as a whole (Fig. 3b). Journals without a WOS category were not well-represented in the hospital collections (Fig. 3b), and WOS categories of journals in the clinical genomics dataset did not vary greatly between institutions, further indicating that hospital collections do not appear to be greatly specialized (Fig. 3b). We measured whether hospital holdings could complement university holdings to increase the proportion of the clinical interpretation dataset covered. However, we found that hospital holdings did not significantly augment university holdings for clinical genomics (Fig. 3c).

## DISCUSSION

We have compiled a large dataset of publications accessed for clinical interpretation of genomic biomarker testing and used it to assess the accessibility of this literature for the health care providers that produce and consume these test results. We find that while 44% of the literature needed is available through open access policies, as much as 56% is not, and providers must rely on institutional library subscriptions to gain complete access. We also find that while university collections were largely sufficient to gain access to all of the literature required, the collections of individual hospital systems covered far less, exposing a gap in literature access that may affect the practice of personalized oncology.

Digital subscription access to journal articles is essential for clinical laboratories because of the need for fast turnaround and high throughput. Clinical labs require a degree of scale to operate economically, and even smaller academic labs issue thousands of reports per year. The time spent per case on interpretation in the clinical setting rarely exceeds an hour, and by necessity must be on the order of minutes for most cases. Interlibrary loan (ILL) has been a key part of hospital libraries' ability to offer access to journals beyond their immediate collections, and digital services like the National Library of Medicine's DOCLINE have enabled fast turnaround for interlibrary requests for medical articles [33]. However, while rapid compared to other ILL services, turnaround times for DOCLINE can still average 24 hours or more, which is incompatible with the needs of clinical labs.

However, ILL may be a useful tool for clinical labs in interpretation of hereditary testing, a related but distinct area of genomic medicine. While personalized cancer biomarker testing requires reporting on all variants of possible clinical significance (which for larger panel tests can number in the dozens), testing for hereditary disease seeks to identify one or a few genomic variants that are causative and diagnostic for a genetic condition. As a result, hereditary reporting is more detailed, and full reporting of all available literature is crucial. Hereditary testing is also generally done on a longer turnaround (typically one month or more as opposed to 1-3 weeks), a timeframe for analysis and reporting that is more compatible with the time needed to fill ILL requests. Additionally, the fees associated with ILL are likely to be lower per case with hereditary testing, with fewer requests required overall.

The collections of academic hospital systems, while smaller than university collections, are an important mechanism in providing access to literature for personnel that do not have access to university resources. While physician staff at the academic hospitals we surveyed generally have a university affiliation, there are many unaffiliated health care providers that interact with precision oncology reports who rely on internal hospital access. Most notably, the work of literature searching on the significance of genomic variants by clinical labs is often done by analysts or technicians who do not have university affiliations and may lack access to paywalled literature. With different cost and resource sharing models between academic medical centers and affiliated universities, the need for hospital collections to provide access to non-physician personnel may differ greatly by institution.

As personalized medicine expands in scope and more decisions are made based on these individualized lab findings, the need for access will certainly extend beyond university-affiliated institutions. Increasingly, generalist and specialist physicians at community hospitals without academic affiliations are using personalized genomics results in cancer care, especially where care is less centralized, such as in rural areas. The need for information access will also be felt acutely in middle-income countries, where precision cancer care is becoming available, but where information infrastructure may be less extensive.

Acquisitions by hospital libraries can be targeted to areas of specific need not covered by general university collections. Such an endeavour would suggest a need for clear communication of needs between libraries and the groups they are serving to understand what sorts of information is used and how it is used by their library's patrons. We assessed whether this could be applied to precision genomics, where coverage of highly specialized publications may be of great value. However, we found that targeted acquisition is not likely to be effective for genomics interpretation. The journals used for genomics interpretation were as likely to be of broad interest as to be focused and did not discernably represent any one subject category. While non-English language journals were frequently not covered by university collections these do not represent a clinically significant group, as they are rarely cited or considered in clinical reporting. Our study does not point to any category of publication that could be selectively acquired to enhance the practice of precision medicine.

While targeted acquisition and partnerships with university libraries can help close the gaps, ultimately staff and resources for hospital libraries remain essential. In May 2021, the Canadian Health Library Association released a statement on the importance of hospital libraries. In it they cited that hospital library resource and workers aid in making better informed clinical decision making, preventing adverse events, and reducing unnecessary treatments, referrals and lengths of stay. However, the trends of library closures, budget reduction, and termination or redeployment of employees has threatened the existence of hospital libraries. [34] Any changes in shared literature access models between hospitals and universities should consider the important role that hospital libraries play in healthcare.

We found that open access policies are an important component of access for the practice of clinical genomics, with nearly half of the publications needed for interpretation available under Gold or Delayed Open Access policies. While hybrid open access journals made up a significant proportion of our dataset, the uptake of open access in this model is generally quite low [35–37], and the overall proportion of truly open-access articles is also likely to be near 50%. The proportion of journals providing delayed open access is especially notable – this class of journal makes up a large segment of this collection of clinically important sources, a segment for which open access can be directly linked to policies put in place by funding agencies like the National Institutes of Health and the Canadian Tri-Council to require that publications resulting from public funding be made accessible [19,38]. Our results show a direct impact of these policies on clinical practice.

Our study focused on Canadian institutions. However, academic medical centers in the US and other countries also routinely use university affiliation to grant access to information resources, and the issues that we explore in this study apply generally in academic medicine. It is important to note that commercial labs have a much larger role in the US than in Canada. Genomic testing for cancer care that is funded by the Canadian public system has largely been carried out in Canadian academic centers, with few patients getting testing from private labs. In contrast, in the US a large proportion of genomic testing is carried out by commercial labs, and a large proportion of patients receive care in non-academic settings. Commercial laboratories must negotiate their own literature access and use a variety of models, but ultimately open access policies benefit these providers and their patients as well.

Another limitation of this study is that while we were able to completely assess current subscription access at universities and health science libraries, we were not able to fully assess whether the full back catalog was available under subscription access in some cases. Our analysis assumes that all back issues are available, but access to older publications may represent an another important constraint on information for genomic reporting.

While this study has focused on the needs of health care providers, the greatest coming challenges around information access in cancer care will likely center on patients. While it has long been agreed that patients have a right to access their medical records, the trend toward patient-centered clinical reporting has accelerated greatly. With the passage of the 21st Century Cures Act in April 2021, patients in the US gained definitive access to the complete narrative text of electronic laboratory and pathology reports, making patients a legally defined part of the audience for laboratory interpretation of genomics results[39]. Along with expanded access to genomics reports and the interpretations on them, patients will need, and demand expanded access to the data underlying those interpretations, and importantly, their providers will increasingly be called on to explain the results of genomic medical tests to them.

## Data Availability

Data associated with this article are available in the Zenodo repository (zenodo.org). DOI: 10.5281/zenodo.7411072.

## SUPPLEMENTAL FILES

**Appendix A:** Examples of interpretive text provided on clinical reports for three genomic tumor variants

**Appendix B:** Scripts used in data analysis
