# Supplementary material for: Clinical reporting for personalized cancer genomics requires extensive access to subscription-only literature
Source: J Med Libr Assoc. 2023 Apr 21;111(1-2):579–90. doi: 10.5195/jmla.2023.1572 (PMC10259627; doi:10.5195/jmla.2023.1572)
Supplement: Supplementary file 1 — Appendix A: Examples of interpretive text provided on clinical reports for three genomic tumor variants [file jmla-111-1-2-579-s01.pdf]

Appendix A: Examples of interpretive text provided on clinical reports for three genomic tumor variants

Example 1:

BRCA1 (NM_007294.4): c.3756_3759delGTCT (p.Ser1253fsTer10)
BRCA1 encodes the protein Brca1, which is involved in the maintenance of genomic stability, including DNA repair, cell cycle checkpoint, and chromosome segregation [PMID:20400477]. Inactivating mutations of BRCA1 can lead to the inability to repair DNA damage and loss of cell cycle checkpoints, which can lead to tumorigenesis [PMID:24638981, PMID:28079255]. Germline mutations in BRCA1 lead to an increased risk of breast, ovarian, gastric, and pancreatic cancers [PMID:22469508, PMID:19454451, PMID:16369438]. The risk of developing breast or ovarian carcinoma in BRCA1 mutation carriers has been reported to be 65-72% and 39-44%, respectively [PMID:12677558, PMID:31650727, PMID:28632866].

The BRCA1 p.Ser1253fsTer10 frameshift variant is predicted to result in an absent or disrupted protein. Pathogenic germline mutations in BRCA1/2 genes are associated with increase the risk for breast and/or ovarian cancer (PMID: 9145678, 16950820) and increased susceptibility to additional cancer types (PMID: 12097290, 12569143, 25356972, 17301269, 18445692, 20587410). The BRCA1 p.Ser1253fsTer10 variant has been reported in the germline context as pathogenic (ClinVar).

Example 2:

TP53 (NM_000546.6): c.613T>G (p.Tyr205Asp)
The TP53 gene encodes the tumor suppressor p53, a protein that is involved in the DNA damage cell cycle checkpoint and causes cell cycle arrest when it senses DNA damage. p53 can also activate DNA repair genes, or induce apoptosis in the presence of DNA damage. Loss of p53 is common in aggressive advanced cancers [PMID:19935675]. The TP53 p.Tyr205Asp missense variant is predicted to be transcriptionally non-functional (IARC TP53 database). Experimental studies suggest another variant at this residue (p.Tyr205Cys) results in reduced transactivation activity (PMID: 15037740, 20505364, 16861262).

Example 3:

EML4 (NM_019063.5: Exon 1) - NRG4 (NM_138573.4: upstream sequence) Fusion
Fusion transcripts were detected that are predicted to represent a fusion between the EML4 and NRG4 genes. The fusion transcripts join the first exon of EML4 with sequences upstream of the NRG4 gene, with some support for transcripts incorporating NRG4. Orientation of these transcripts is such that the predicted protein fusion would join the first exon of EML4 to all or most of the NRG4 protein. It is predicted that is fusion protein would be under control of the EML4 promoter. While EML4-NRG4 fusions have not been described previously, fusions involving NRG1, a protein related to NRG4, have been characterized in multiple tumor types (PMID: 34098222).

EML4 is a fusion partner in driver fusions found in multiple cancers, most notably in the EML4-ALK fusion found recurrently in non-small cell lung cancer. EML4 is the 5' partner in these recurrent fusions, and it is thought that EML4 contributes to oncogenicity by putting the fusion partner under control of a broadly expressed promoter (PMID: 29327716). The fusion transcripts detected in this sample, which incorporate only the first exon of EML4, are consistent with this potential oncogenic mechanism.

NRG4 encodes a member of the neuregulin family of proteins, which are extracellular ligands that can activate the ERBB family of growth factor receptors. Fusions involving NRG4 have been reported previously in ovarian cancer but have not been functionally characterized (PMID: 29633253). NRG4 primarily activates ERBB4, a receptor tyrosine kinase of the ERB/HER family. ERBB4 signals as a homodimer or heterodimer with ERBB2, activating multiple downstream pathways to regulate growth (PMID: 28791631, 34885957). While targeting of ERBB4 has not been studied clinically, some small molecule inhibitors targeting the ERB family have been found to have activity against ERBB4 in vitro (PMID: 34885957).
