# Supplementary material for: Clinical reporting for personalized cancer genomics requires extensive access to subscription-only literature
Source: J Med Libr Assoc. 2023 Apr 21;111(1-2):579–90. doi: 10.5195/jmla.2023.1572 (PMC10259627; doi:10.5195/jmla.2023.1572)
Supplement: Supplementary file 2 — Appendix B: Scripts used in data analysis [file jmla-111-1-2-579-s02.pdf]

Appendix B: Scripts used in data analysis

---

1. Syntax for retrieval of Pubmed IDs from Watson for Genomics JSON output files.

```
#!/bin/bash

grep -iE "PubmedID|evidence|PMID"  ../TST15/TST15_Watson_DOWN_874/UHN-TST15-
0*.json  | cut -f 3 -d : | tr -d '"' | sort -u > tst15.pmids
```

---

2. Python script for retrieval of PMID dates and journal names via PubMed API

```
#!/usr/bin/env python3

import os
import sys
import argparse
import re
import tempfile
#from string import Template
#from xlrd import open_workbook
#from docx import Document
#from docx.shared import Pt
#from docx.enum.text import WD_ALIGN_PARAGRAPH
from time import sleep
import datetime
from datetime import timedelta

def init():
    '''
    Initialization method to get command line parameters.
    '''
    parser = argparse.ArgumentParser(
        description='This script compares PMIDs.')

    parser.add_argument('-p', '--pmids', required=True,
                        help='input file.')
    parser.add_argument('-d', '--dates', required=True,
                        help='input file.')
    parser.add_argument('-g', '--get', action='store_true', required=False,
help='download dates')

    options = parser.parse_args()
    return options

def main(args):

    dates = {}
    with open(args.dates, 'r') as f:
        for line in f:
            #print(line.rstrip())
            pmid, year, month, day = line.rstrip().split(' ')
            #print(pmid, 'y', year, 'm', month, 'd', day)
```

```
        if day == '' or day == 'None':
            day = 15
        if month == '' or month == 'None':
            month = 7
        #print(pmid, year, month, day)
        dates[pmid] = datetime.datetime(int(year), int(month), int(day))

pfile = args.pmids

allamdl = set()
allwatson = set()
aaamdl = []
aawatson = []
with open(pfile, 'r') as f:
    for line in f:
        #print(line.rstrip())
        amdl, watson = line.rstrip().split(',')
        amdl = amdl.split(' ')
        [allamdl.add(x) for x in amdl if x != '']
        [aaamdl.append(x) for x in amdl if x != '']
        watson = watson.split(' ')
        [allwatson.add(x) for x in watson if x != '']
        [aawatson.append(x) for x in watson if x != '']

        # if len(amdl) == 0 and len(watson) == 0:
        #     print('oops', line.rstrip())
        #     continue
        allids = set()
        for a in amdl:
            allids.add(a)
        for w in watson:
            allids.add(w)
        acount = 0
        wcount = 0
        bcount = 0
        adate = []
        wdate = []
        for i in allids:
            if i == '':
                continue
            if i in amdl:
                acount += 1
                adate.append(dates[i])
            if i in watson:
                wcount += 1
                wdate.append(dates[i])
            if i in amdl and i in watson:
                bcount += 1
        #if bcount > 0:

        aavg = 0
        wavg = 0
        asum = sum(map(datetime.datetime.timestamp,adate))
        wsum = sum(map(datetime.datetime.timestamp,wdate))
        try:
            aavg = datetime.datetime.fromtimestamp(asum/len(adate))
        except ZeroDivisionError:
```

```
            pass
        try:
            wavg = datetime.datetime.fromtimestamp(wsum/len(wdate))
        except ZeroDivisionError:
            pass
        print(acount, aavg, wcount, wavg, bcount, len(allids))

print('all amdl', len(allamdl))
print('all watson', len(allwatson))
inter = allamdl.intersection(allwatson)
#print('inter is')
#print(inter)
print('intersection', len(inter))
adate = []
for i in allamdl:
    adate.append(dates[i])
wdate = []
for i in allwatson:
    wdate.append(dates[i])
adate.sort()
wdate.sort()
asum = sum(map(datetime.datetime.timestamp,adate))
wsum = sum(map(datetime.datetime.timestamp,wdate))
try:
    aavg = datetime.datetime.fromtimestamp(asum/len(adate))
except ZeroDivisionError:
    pass
try:
    wavg = datetime.datetime.fromtimestamp(wsum/len(wdate))
except ZeroDivisionError:
    pass
print('amdl average', aavg)
print('amdl median', adate[int(len(adate)/2)])
print('amdl oldest', adate[0])
print('amdl most recent', adate[-1])
print('watson average', wavg)
print('watson median', wdate[int(len(wdate)/2)])
print('watson oldest', wdate[0])
print('watson most recent', wdate[-1])
idate = []
for i in inter:
    idate.append(dates[i])
idate.sort()
isum = sum(map(datetime.datetime.timestamp,idate))
try:
    iavg = datetime.datetime.fromtimestamp(isum/len(idate))
except ZeroDivisionError:
    pass
print('intersection average', iavg)
print('intersection median', idate[int(len(idate)/2)])

adate = []
for i in aaamdl:
    adate.append(dates[i])
wdate = []
for i in aawatson:
    wdate.append(dates[i])
```

```
    adate.sort()
    wdate.sort()
    asum = sum(map(datetime.datetime.timestamp,adate))
    wsum = sum(map(datetime.datetime.timestamp,wdate))
    try:
        aavg = datetime.datetime.fromtimestamp(asum/len(adate))
    except ZeroDivisionError:
        pass
    try:
        wavg = datetime.datetime.fromtimestamp(wsum/len(wdate))
    except ZeroDivisionError:
        pass
    print('all amdl average', aavg, len(adate))
    print('all amdl median', adate[int(len(adate)/2)])
    print('all watson average', wavg, len(wdate))
    print('all watson median', wdate[int(len(wdate)/2)])


def get_dates(args):
    from pubmed_lookup import PubMedLookup
    from pubmed_lookup import Publication
    pfile = args.pmids
    ids = set()
    have = set()
    with open(args.dates, 'r') as f:
        for line in f:
            have.add(line.split('\t')[0])

    # print('have', len(have))

    with open(pfile, 'r') as f:
        for line in f:
            line = line.rstrip()
            if line not in have:
                ids.add(line)

    email = 'gdowns@uhnresearch.ca'
    base = 'http://www.ncbi.nlm.nih.gov/pubmed/'
    # print('ids has', len(ids))
    # sys.exit()

    for pmid in ids:
        if pmid == '':
            continue
        # print(pmid)
        sleep(2)
        lookup = PubMedLookup(base + str(pmid), email)
        try:
            publication = Publication(lookup)
        except IndexError:
            print(str(pmid), 'IndexError')
            continue
        except ConnectionResetError:
            # print(str(pmid), 'ConnectionResetError')
            try:
                publication = Publication(lookup, resolve_doi=False)
            except:
```

```
                print(str(pmid), 'Error', sys.exc_info()[0])
                continue
        except:
            print(str(pmid), 'Error', sys.exc_info()[0])
            continue
        print('\t'.join([str(pmid), str(publication.year),
str(publication.month), str(publication.day), str(publication.journal)]))
        #print(str(pmid), publication.journal)

if __name__ == '__main__':

    args = init()
    if args.get:
        get_dates(args)
    else:
        main(args)
```

---

3. Python script for compilation of data

```
#!/usr/bin/env python3

import os
import sys
import argparse
import re
import tempfile
from time import sleep
import datetime
from datetime import timedelta
import numpy as np

def init():
    '''
    Initialization method to get command line parameters.
    '''
    parser = argparse.ArgumentParser(
        description='This script compares PMIDs.')

    parser.add_argument('-p', '--pmids', required=True,
                        help='input file.')
    parser.add_argument('-j', '--journals', required=True,
                        help='input file.')

    options = parser.parse_args()
    return options

if __name__ == '__main__':

    args = init()

    p2j = {}
    j2p = {}
    jtotal = {}

    with open(args.journals, 'r') as f:
```

```
        for line in f:
            # print(line)
            pmid, d1, d2, d3, journal = line.rstrip().split('\t')
            p2j[pmid] = journal


    # cat agile.pmids.csv | tr , '\n' | tr ';' '\n' | tr  ' ' '\n' | grep -vE
"^$" | sort -n > agile.all.citations
    with open(args.pmids, 'r') as f:
        for line in f:
            pmid = line.rstrip()
            journal = p2j[pmid]
            if journal not in jtotal:
                jtotal[journal] = 1
            else:
                jtotal[journal] += 1
            if journal not in j2p:
                j2p[journal] = {}
            if pmid not in j2p[journal]:
                j2p[journal][pmid] = 1
            else:
                j2p[journal][pmid] += 1

    for j in sorted(j2p):
        pcount = 0
        counts = []
        for p in j2p[j]:
            pcount += 1
            counts.append(int(j2p[j][p]))
        print('\t'.join([j, str(jtotal[j]), str(pcount), str(np.min(counts)),
str(round(np.mean(counts), 4)), str(np.max(counts))]))
        # print(j, jtotal[j], pcount, counts)
```
